# Intentions to Perform Non-Pharmaceutical Protective Behaviors during Influenza Outbreaks in Sweden: A Cross-Sectional Study following a Mass Vaccination Campaign

**DOI:** 10.1371/journal.pone.0091060

**Published:** 2014-03-07

**Authors:** Toomas Timpka, Armin Spreco, Elin Gursky, Olle Eriksson, Örjan Dahlström, Magnus Strömgren, Joakim Ekberg, Sofie Pilemalm, David Karlsson, Jorma Hinkula, Einar Holm

**Affiliations:** 1 Department of Public Health, Östergötland County Council, Linköping, Sweden; 2 Department of Medical and Health Sciences, Linköping University, Linköping, Sweden; 3 National Strategies Support Directorate, ANSER/Analytic Services Inc, Arlington, Virginia, United States of America; 4 Linnaeus Centre HEAD, Department of Behavioural Sciences, Linköping University, Linköping, Sweden; 5 Department of Geography and Economic History, Umeå University, Umeå, Sweden; 6 Department of Clinical and Experimental Medicine, Linköping University, Linköping, Sweden; Alberta Provincial Laboratory for Public Health/University of Alberta, Canada

## Abstract

Failure to incorporate the beliefs and attitudes of the public into theoretical models of preparedness has been identified as a weakness in strategies to mitigate infectious disease outbreaks. We administered a cross-sectional telephone survey to a representative sample (n = 443) of the Swedish adult population to examine whether self-reported intentions to improve personal hygiene and increase social distancing during influenza outbreaks could be explained by trust in official information, self-reported health (SF-8), sociodemographic factors, and determinants postulated in protection motivation theory, namely threat appraisal and coping appraisal. The interviewees were asked to make their appraisals for two scenarios: a) an influenza with low case fatality and mild lifestyle impact; b) severe influenza with high case fatality and serious disturbances of societal functions. Every second respondent (50.0%) reported high trust in official information about influenza. The proportion that reported intentions to take deliberate actions to improve personal hygiene during outbreaks ranged between 45–85%, while less than 25% said that they intended to increase social distancing. Multiple logistic regression models with coping appraisal as the explanatory factor most frequently contributing to the explanation of the variance in intentions showed strong discriminatory performance for staying home while not ill (mild outbreaks: Area under the curve [AUC] 0.85 (95% confidence interval 0.82;0.89), severe outbreaks AUC 0.82 (95% CI 0.77;0.85)) and acceptable performance with regard to avoiding public transportation (AUC 0.78 (0.74;0.82), AUC 0.77 (0.72;0.82)), using handwash products (AUC 0.70 (0.65;0.75), AUC 0.76 (0.71;0.80)), and frequently washing hands (AUC 0.71 (0.66;0.76), AUC 0.75 (0.71;0.80)). We conclude that coping appraisal was the explanatory factor most frequently included in statistical models explaining self-reported intentions to carry out non-pharmaceutical health actions in the Swedish outlined context, and that variations in threat appraisal played a smaller role in these models despite scientific uncertainties surrounding a recent mass vaccination campaign.

## Introduction

Although encouraging the public to undertake specific protective behaviors has proved useful in containing outbreaks of infectious disease [1], more research has been called for examining the social, demographic, and cultural factors that influence these efforts [2]. This is particularly important to understanding people’s hesitations to heed official advice, particularly in the absence of clear scientific evidence regarding the disease outbreak [3]. The AS03-adjuvanted Pandemrix® was the most commonly used vaccine in response to the Influenza A(H1N1)pdm09 outbreak in Europe [4]; Finland and Sweden recommended this vaccine to their entire populations. In August 2010 reports of a possible association between exposure to the vaccine and occurrence of narcolepsy in children and adolescents emerged in both the latter countries, which led to a review of the vaccine by the European Medicines Agency (EMA). Subsequently, increased narcolepsy diagnoses associated with the start of the campaign have been confirmed [5]. In Sweden, scientific uncertainty regarding the safety of this mass vaccination was both publicly discussed [6] and questioned by researchers [7].

Beliefs that the interventions suggested are effective and safe [8], that the illness has severe consequences [9], and that there is a high likelihood of exposure [10] have been associated with compliance with behavioral recommendations. It has also been pointed out that behavioral research in epidemics should not only identify determinants of individual and population behavioral responses, but also clarify the mechanisms underpinning these [11]. Protection Motivation Theory (PMT) [12], [13] posits that an intention to perform protective activities is determined by perceptions of threat and the ability to cope. In addition to intentions and preceptions, a recent review concluded that protective behavior needs to be investigated with regard to sociodemiograpic characteristics in order to identify the “contagious” effect and contextual nature of perceptions and mediating mechanisms [14]. For instance, coping appraisals are made in interaction with environmental resources, which vary in availability across population subgroups. Protective behavior associated with influenza outbreaks has also been investigated with regard to general estimates of health status [15], but few studies have used validated measures of self-rated health as a means for the sub categorization. At present, several such measures are available for use in population-based research [16].

To provide a snapshot of intended self-protective behaviors during a period when scientific uncertainty pervaded public discussions addressing infectious disease control, we carried out a cross-sectional telephone survey of a demographically representative sample of the Swedish population. The specific aim was to examine to what extent self-reported intentions to improve personal hygiene and increase social distancing during influenza outbreaks can be explained by perceptions of threat and the ability to cope as outlined in PMT, self- reported assessments of health, trust in official information, and sociodemiographic factors.

## Methods

The study used a cross-sectional design to analyze associations between intended protective behaviors during influenza outbreaks and items in a theoretical model of explanatory factors [14], [17]. A random sample of 1,011 persons ranging between 20–90 years of age was drawn from the Swedish national population register. A combined telephone and questionnaire survey was carried out during the first quarter of 2012.

### Ethics Statement

The study was conducted according to the World Medical Association’s Declaration of Helsinki from 1964 regarding ethical principles for medical research involving human subjects, revised in 2008. Potential study sample participants were informed about the study by letter via postal mail and invited to participate in a telephone survey on protective behaviors during influenza outbreaks. Those agreeing to participate returned their consent in writing. All collected data were managed confidentially and analyzed anonymously. The study design was approved by the institutional (ethics) review board at Umeå University (Dnr 2011-314-31Ö).

### Theoretical Model

A hypothetical explanatory model was constructed to inform the analysis of the main research question; i.e. to what extent self-reported intentions to perform protective behaviors during influenza outbreaks can be explained by perceptions of threat and the ability to cope as outlined in the PMT, self-assessments of health status, trust in official information, and sociodemiographic factors. In this model, protective behaviors during outbreaks are restricted to two categories: increased personal hygiene (use of disinfectants and other handwash products; frequent washing of hands when having touched common objects, such as door knobs) and social distancing (staying home from work or school; avoiding use of public transportation). The intentions to carry out a protective behavior are assessed by asking whether the respondent would try to perform the behavior during a mild and severe influenza outbreak, respectively. Both outbreak scenarios described personal risk of infection as high (i.e., 1 in 3 people infected). The mild influenza description details moderate health consequences (less than 1 in 1000 infected people dying) and a minor lifestyle impact (services mainly operating normally). The severe scenario describes serious health consequences (1 in 50–100 infected people dying) and services no longer being able to operate normally.

The first set of explanatory factors concerned perceptions of threat and the ability to cope. Based on the notion of subjective expected utility [18], which postulates that people’s choices are a product of assessments of probability and utility of options, health-related methodologies such as the PMT and the Health Belief Model [19] have included formally quantified models of subjective health risk perceptions, i.e., as the likelihood of contracting a disease multiplied by disease severity. Together with different types of cost–benefit valuations and self-efficacy expectations, these perceptions of risk are presumed to determine health-protective behaviors. In the present study, the collection and analysis of data on protection motivation in relation to influenza outbreaks are structured according to the PMT. This theory suggests that threat appraisal will generate an intention to act, while coping appraisal determines the type of action. Threat appraisal is in this study characterized in its three dimensions [11], [20]–[21]:

perceived relative risk of catching influenza; measured by one item assessing personal likelihood of infection, if no preventative action was taken,
anxiety about catching mild and severe influenza; measured by one item for each influenza type, and
perceived severity of the consequences of catching mild and severe influenza; measured by one item for each influenza type.

Coping appraisal is also represented in its three dimensions:

Response efficacy; assessed by one item asking about protecting oneself from influenza by employing enhanced personal hygiene and one item asking about social distancing,
Self-efficacy; measured by two items asking whether the respondent felt it is possible to carry out protective behaviors by social distancing and increased personal hygiene, respectively, and whether they were confident they could carry out these actions if they so desired [22], and
Response costs; defined as the estimated efforts needed to overcome perceived barriers on carrying out protective actions. For social distancing, this dimension was assessed by asking for ‘work concerns’, i.e. guilt and anxiety about not completing work. Response costs for increased personal hygiene were assessed through items asking for concerns associated with acquiring adequate soaps and disinfectants (handwash products) and learning the correct techniques to use them.

Self-reported health assessments have in epidemiological studies been found to be valid indicators of health status as measured by prediction of future physician contacts and all-cause mortality [23]. In this study, self-reported health is measured by the SF-8™ 24-hour recall questionnaire in order to examine associations with intentions to carry out protective behaviors. This general self-reported health instrument contains eight health-related questions that, in turn, can be summarized in two overall measures of physical and mental health: physical component summary (PCS) and mental component summary (MCS), respectively [24]. It is derived from the SF-36 for the purposes of yielding comparable scores for the 8 health dimensions and 2 summary measures of the SF-36 with minimal respondent burden.

Trust in government information during influenza outbreaks has in previous studies been found to be associated with greater self-efficacy and personal hygiene [25]. Trust in official information was therefore included in the explanatory model, asking for agreement with a single statement about trust in government information during outbreaks. The sociodemiographic factors included in the model were marriage status, number of children living at home, formal education, employment status, and ethnicity.

### Data Collection

Prior to the telephone call, the subjects were asked to complete a paper-based survey, querying for sociodemiographic data and data elements from the SF-8™. The remaining data were collected in the telephone interview. To catalyze their considerations about the research topics, each subject was presented with brief scenarios of mild and severe influenza outbreaks. Interview data were derived from open statements, and the respondents were asked to score their agreement along a seven-point scale from 1 (*strongly disagree*) to 7 (*strongly agree*). The collection of data on perceptions associated with precautionary behaviors was structured in accordance with the PMT (Text S1). To assess trust in official information in this study, the single statement “For information during influenza outbreaks I do rely on government sources” was used.

### Data Analyses

We conducted a drop-out analysis based on the demographic variables available for the entire sample, i.e. gender, age and place of residence. All collected data were first subjected to descriptive statistics, i.e. mean, median and standard deviation for continuous data and frequency and proportions (%) for categorical data. The primary end points for the ensuing analyses were intentions to increase social distancing (staying home while not ill; avoid public transportation), and enhance personal hygiene (use of handwash; frequent washing of hands after touching common objects) during mild and severe influenza outbreaks, respectively. The theoretical model of potential explanatory factors was used as the basis for the analysis. For each endpoint, logistic regression analyses were applied using the items in the model as explanatory variables. These included trust in official information; variables corresponding to PMT items (the threat appraisal items of perceived personal risk, emotional response (worry), perceived severity; and the coping appraisal items of general response efficacy, self-efficacy, and response costs); variables representing the SF-8 summary items (PCS and MCS); and sociodemographic characteristics (age, gender, educational level, living with partner, living with child, and employment). When used as response variables, ordinal variables were dichotomized (agree/do not agree). To contrast expected perceptions against other perceptions, the variables were converted with the agreement scores in the expected extreme as one category. For threat appraisal, agreement scores in the low extreme were contrasted against other opinions, except for the estimates of the severity of the consequences of getting infected where the scores in the high extreme were contrasted against the other opinions. Regarding coping appraisal, the personal hygiene scores in the high extreme were contrasted against other opinions for response efficacy and self-efficacy and in the low extreme for response costs. For social distancing, agreement scores in the low extreme were contrasted against other opinions for response efficacy and in the high extreme for self-efficacy and response costs.

The area under the ROC curve (AUC) was used as model performance indicator and Nagelkerke R^2^ to estimate the determination level for each model. The limits for interpreting the AUC (or c-statistic) were set to 0.90, 0.80, and 0.70, denoting very strong (outstanding), strong (excellent), and acceptable discriminatory performance, respectively [26]. All tests were two-sided and *P*<0.05 was regarded as statistically significant. All calculations were done using SPSS version 18 or higher.

## Results

Two-hundred and fifty-four persons in the total population sample (n = 1,011) could not be reached by a telephone call. Of the 757 persons reached, 443 provided a complete response, leading to a 59% response rate to the telephone survey and a 44% participation rate with regard to the total sample. The age category 65–90 years was slightly over-represented (54% response rate) among the study participants when compared to the total population sample (p = 0.039). However, the effect size of this difference in participation was small (Cramer’s V = 0.08). Thus, while elderly individuals were overrepresented in our data, the impact of this deviation from the reference population was of a small magnitude. In terms of place of residence, those living in small labor market regions (with a total population of less than 100,000 inhabitants) exhibited the highest participation rates: 51%, compared to 41% in large regions (with a population greater than 1 000,000 inhabitants). The basic sociodemiographic characteristics of the final study participants are displayed in Table 1. The general level of health in the study population as measured by SF-8 scores was above the reference values for all items except for Physical functioning and Vitality (lower scores) and General Health (equal scores) (Table 2). There was no statistically significant difference between men and women regarding the mean scores of any SF-8 item or summary component.

**Table 1:** Sociodemiographic characteristics of of the study population (n = 443).

	Men n = 222	Women n = 221	Total
Mean age years (s.d.)	50.9 (17.7)	51.9 (17.7)	51.4 (17.7)
Academic education n (%)	95 (41.3)	101 (47.2)	196 (44.1)
Born abroad n (%)	33 (14.3)	23 (10.7)	56 (12.6)
Lives with partner n (%)	168 (71.5)	132 (61.1)	300 (66.5)
children n (%)	84 (35.7)	79 (36.6)	163 (36.1)
Employed/student n (%)	154 (67.0)	120 (56.1)	274 (61.7)

**Table 2:** Self-reported health of the study population displayed by the mean values (95% C.I.) on subscales and summary components of the Short Form-8 (SF-8) (n = 443).

	Mean	95% CI	Reference
General Health	48.95	48.13–49.77	48.90
Physical functioning	49.41	48.61–50.21	49.30
Role Physical	50.73	50.04–51.42	49.40
Bodily pain	53.74	52.87–54.60	50.00
Vitality	49.11	48.33–49.89	50.00
Social functioning	52.02	51.32–52.72	49.30
Mental health	52.39	51.68–53.09	49.10
Role emotional	50.02	49.45–50.59	48.90
Physical component summary (PCS)	50.98	50.09–51.87	49.40
Mental component summary (MCS)	52.52	51.78–53.26	49.40

### Trust in Official Information Sources

Every second respondent (50.0%) reported high or very high trust (scores 6–7) in information about influenza provided by official sources (mean score 5.3; median 5.5; Standard Deviation (SD) 1.7)). Neither age, education, employment nor any component of self-rated health was associated with trust in official information about influenza. However, the level of trust was associated with gender, with men reporting lower trust levels than women (p = 0.018; Odds Ratio (OR) 0.60 (95% Confidence Interval (CI) 0.40;0.91)).

### Intended Protective Behavior during Influenza Outbreaks

Regarding social distancing measures, 9% of the respondents scored strong (strong or very strong) agreement with the stated intention to stay home when not ill during mild influenza outbreaks, and 11% of the respondents scored strong agreement with this intent during severe outbreaks. More than twice as many respondents (23%) scored strong agreement with avoiding use of public transportation during a mild outbreak, while 29% of the respondents scored strong agreement with this intention during a severe outbreak. Regarding measures related to personal hygiene, 77% of the respondents scored strong agreement with the stated intention to use handwash products during mild outbreaks, while 85% of the respondents scored strong agreement with this intention during severe outbreaks. Regarding the intention to frequently engage in handwashing, 46% reported strong agreement in association to mild influenza outbreaks and 60% in association to severe outbreaks.

### Factors Associated with Intended Protective Behavior during Mild and Severe Outbreaks

A model describing the intention to stay home without being ill during a mild influenza outbreak included eight significant variables and displayed a strong discriminative performance (AUC 0.85 (95% CI 0.82;0.89)) (Table 3). This self-reported intention was strongly associated with coping appraisal; low perceived response costs associated with staying home and self-efficacy with regard to social distancing; and, interestingly, to a disbelief in the general efficacy of social distancing as an infectious disease control measure. Planning to stay home was also strongly associated with male gender and, with a weaker association, to being unemployed and living with a partner. The intention was also associated with threat appraisal, although with a weaker strength; with worry about getting infected and high perceived severity of the influenza threat. In comparison, the intention to stay home without being ill during a severe outbreak was represented by a model including only four significant variables, but that also displayed a strong discriminative performance (AUC 0.82 ((95% CI 0.77;0.85)). As for the mild outbreak scenario, this intention was strongly associated with coping appraisal; to response costs and perceived self-efficacy with regard to social distancing. However, staying home during a severe outbreak was also strongly associated with threat appraisal related to concerns about getting infected. Regarding sociodemographic factors, this intention was only associated with not having employment.

**Table 3:** Simple and multiple logistic regression models of explanatory factors for the intention to stay home without being ill displayed by influenza outbreak scenario.

	Mild influenza scenario		Severe influenza scenario	
	Simple models	Multiple model^a^	Simple models	Multiple model^b^
	OR (95% C.I.)	OR (95% C.I.)	OR (95% C.I.)	OR (95% C.I.)
**Trust in information**				
Trust in official sources	n.s.	n.s.	n.s.	n.s.
**Threat appraisal**				
Perceived personal risk	1.28** (1.13–1.44)	n.s.	1.24** (1.10–1.40)	n.s.
Emotional response to threat	1.34** (1.20–1.51)	1.19* (1.01–1.40)	1.40** (1.25–1.57)	1.35** (1.16–1.56)
Perceived severity of health threat	1.27** (1.14–1.42)	1.27* (1.10–1.48)	1.19** (1.07–1.33)	n.s.
**Coping appraisal**				
General response efficacy†	1.15* (1.03–1.29)	0.72* (0.58–0.89)	1.18* (1.07–1.32)	n.s.
Self-efficacy‡	1.32** (1.18–1.47)	1.59** (1.29–1.96)	1.31** (1.18–1.45)	1.43** (1.19–1.72)
Response costs‡	0.13** (0.08–0.22)	0.13** (0.07–0.23)	0.17** (0.11–0.25)	0.16** (0.10–0.27)
**Sociodemographic characteristics**				
Age	1.03** (1.01–1.04)	n.s.	1.02* (1.00–1.03)	n.s.
Gender (Male = 1;Female = 0)	1.69* (1.13–2.53)	2.54** (1.48–4.37)	n.s.	n.s.
Higher education§ (Yes = 1;No = 0)	n.s.	n.s.	n.s.	n.s.
Born in the country (Yes = 1;No = 0)	0.45* (0.25–0.79)	n.s.	0.46* (0.26–0.82)	n.s.
Living with partner (Yes = 1;No = 0)	n.s.	1.90* (1.04–3.48).	n.s.	n.s.
Living with child (Yes = 1;No = 0)	0.55* (0.35–0.84)	n.s.	n.s.	n.s.
Employment (Yes = 1;No = 0)	0.31** (0.21–0.47)	0.42* (0.22–0.81)	0.46** (0.31–0.68)	0.52* (0.29–0.95)
**Self-rated health (SF-8)**				
Physical Component Summary (PCS)	0.98* (0.96–1.00)	n.s.	n.s.	n.s.
Mental Component Summary (MCS)	n.s.	n.s.	n.s.	n.s.

n.s. = not statistically significant.

* = statistically significant on p = 0.05 level, ** = statistically significant on p = 0.001 level,

a Specificity = 86.3%, Sensitivity = 63.9%, Correctly classified = 79.0%, Nagelkerke R^2^ = 0.450,

b Specificity = 78.5%, Sensitivity = 67.7%, Correctly classified = 73.8%, Nagelkerke R^2^ = 0.379.

† Item specific for social distancing.

‡ Item specific for intention to stay home.

§ Formal education past high school/secondary school.

The intention to avoid using public transportation during a mild influenza outbreak was represented by a model including six significant variables and an acceptable discriminative performance (AUC 0.78 (95% CI 0.74;0.82)) (Table 4). This self-reported intention was, also, strongly associated with coping appraisal; to perceived response costs associated with avoiding public transportation and to self-efficacy with regard to social distancing. The intention was also strongly associated with threat appraisal in terms of worry about getting infected. In addition, avoiding use of public transportation was associated with a lower level of formal education, living with a partner, and high trust in official information. In contrast, the intention to avoid public transportation during a severe influenza outbreak was described by a model including four significant variables and an acceptable discriminative performance (AUC 0.77 (95% CI 0.72;0.82)). As for the mild outbreak scenario, avoiding public transportation during severe outbreaks was strongly associated with coping appraisal; to response costs; and, with weaker strength, to perceived self-efficacy with regard to personal social distancing. With regard to threat appraisal, avoiding public transportation during a severe outbreak was associated with worry about getting infected and a high perceived severity of the influenza threat.

**Table 4:** Simple and multiple logistic regression models of explanatory factors for the intention to avoid using public transport displayed by influenza outbreak scenario.

	Mild influenza scenario		Severe influenza scenario	
	Simple models	Multiple model^a^	Simple models	Multiple model^b^
	OR (95% C.I.)	OR (95% C.I.)	OR (95% C.I.)	OR (95% C.I.)
**Trust in information**				
Trust in official sources	1.15* (1.03–1.29)	1.15* (1.00–1.31)	n.s.	n.s.
**Threat appraisal**				
Perceived personal risk	1.37** (1.20–1.57)	n.s.	1.32** (1.14–1.53)	n.s.
Emotional response to threat	1.63** (1.39–1.91)	1.46** (1.22–1.76)	1.50** (1.29–1.73)	1.29* (1.10–1.51)
Perceived severity of health threat	1.23** (1.11–1.37)	n.s.	1.27** (1.14–1.42)	1.17* (1.03–1.32)
**Coping appraisal**				
General response efficacy†	1.22** (1.10–1.35)	n.s.	1.28** (1.15–1.43)	n.s.
Self-efficacy‡	1.29** (1.17–1.42)	1.28* (1.08–1.52)	1.32** (1.19–1.47)	1.25* (1.05–1.49)
Response costs‡	0.34** (0.23–0.50)	0.38** (0.24–0.59)	0.35** (0.23–0.54)	0.40** (0.25–0.64)
**Sociodemographic characteristics**				
Age	1.02* (1.00–1.03)	n.s.	1.01* (1.00–1.03)	n.s.
Gender (Male = 1;Female = 0)	n.s.	n.s.	n.s.	n.s.
Higher education§ (Yes = 1;No = 0)	n.s.	0.62* (0.40–0.97)	n.s.	n.s.
Born in the country (Yes = 1;No = 0)	n.s.	n.s.	n.s.	n.s.
Living with partner (Yes = 1;No = 0)	n.s.	1.73* (1.05–2.86)	n.s.	n.s.
Living with child (Yes = 1;No = 0)	n.s.	n.s.	n.s.	n.s.
Employment (Yes = 1;No = 0)	0.50** (0.34–0.74)	n.s.	n.s.	n.s.
**Self-rated health (SF-8)**				
Physical Component Summary (PCS)	n.s.	n.s.	0.97* (0.95–1.00)	n.s.
Mental Component Summary (MCS)	0.97* (0.95–1.00)	n.s.	n.s.	n.s.

n.s. = not statistically significant.

* = statistically significant on p = 0.05 level, ** = statistically significant on p = 0.001 level,

a Specificity = 63.6%, Sensitivity = 74.7%, Correctly classified = 69.8%, Nagelkerke R^2^ = 0.309,

b Specificity = 41.4%, Sensitivity = 89.8%, Correctly classified = 74.5%, Nagelkerke R^2^ = 0.268.

† Item specific for social distancing.

‡ Item specific for intention to use public transport.

§ Formal education past high school/secondary school.

Planning to use handwash products during a mild influenza outbreak was described by a model including three significant variables and an acceptable discriminative performance (AUC 0.70 (95% CI 0.65;0.75)) (Table 5). Planning to use handwash was strongly associated with female gender. This intention was, for mild outbreaks, also explained by self-efficacy with regard to personal hygiene and trust in official information. For the severe outbreak scenario, planning to use handwash products was represented by a model including four significant variables and an acceptable discriminative performance (AUC 0.76 (95% CI 0.71;0.80)). This intention was, too, strongly associated with female gender. In addition, it was strongly associated with coping appraisal; to a belief in the general efficacy of increased personal hygiene; and low response costs associated with acquiring of suitable products. Contrary to any of the other intended behaviors studied, the intention to use handwash products during severe outbreaks was associated with low self-rated physical health.

**Table 5:** Simple and multiple logistic regression models of explanatory factors for the intention to use handwash displayed by influenza outbreak scenario.

	Mild outbreak scenario		Severe influenza scenario	
	Simple models	Multiple model^a^	Simple models	Multiple model^b^
	OR (95% C.I.)	OR (95% C.I.)	OR (95% C.I.)	OR (95% C.I.)
**Trust in information**				
Trust in official sources	1.25** (1.11–1.40)	1.20* (1.06–1.36)	1.18* (1.04–1.34)	n.s.
**Threat appraisal**				
Perceived personal risk	n.s.	n.s.	n.s.	n.s.
Emotional response to threat	n.s.	n.s.	n.s.	n.s.
Perceived severity of health threat	n.s.	n.s.	n.s.	n.s.
**Coping appraisal**				
General response efficacy†	1.27** (1.11–1.46)	n.s.	1.35** (1.17–1.55)	n.s.
Self-efficacy‡	2.23** (1.49–3.33)	2.02* (1.25–3.29)	3.24** (2.00–5.27)	3.25** (1.79–5.91)
Response costs‡	n.s.)	n.s.	n.s.	0.05* (0.01–0.43)
**Sociodemographic characteristics**				
Age	n.s.	n.s.	n.s.	n.s.
Gender (Male = 1;Female = 0)	0.40** (0.27–0.60)	0.46** (0.30–0.70)	0.25** (0.15–0.41)	0.24** (0.14–0.41)
Higher education§ (Yes = 1;No = 0)	n.s.	n.s.	n.s.	n.s.
Born in the country (Yes = 1;No = 0)	n.s.	n.s.	n.s.	n.s.
Living with partner (Yes = 1;No = 0)	n.s.	n.s.	n.s.	n.s.
Living with child (Yes = 1;No = 0)	n.s.	n.s.	n.s.	n.s.
Employment (Yes = 1;No = 0)	n.s.	n.s.	n.s.	n.s.
**Self-rated health (SF-8)**				
Physical Component Summary (PCS)	n.s.	n.s.	0.97* (0.94–1.00)	0.97* (0.94–1.00)
Mental Component Summary (MCS)	n.s.	n.s.	n.s.	n.s.

n.s. = not statistically significant.

* = statistically significant on p = 0.05 level, ** = statistically significant on p = 0.001 level,

a Specificity = 39.3%, Sensitivity = 85.0%, Correctly classified = 68.2%, Nagelkerke R^2^ = 0.157,

b Specificity = 29.6%, Sensitivity = 94.9%, Correctly classified = 79.0%, Nagelkerke R^2^ = 0.379.

† Item specific for personal hygiene.

‡ Item specific for intention to use handwash.

§ Formal education past high school/secondary school.

An intention to frequently engage in handwashing after having touched common objects during a mild influenza outbreak was represented by a model including four significant variables and an acceptable discriminative performance (AUC 0.71 (95% CI 0.66;0.76)) (Table 6). The intention was strongly associated with coping appraisal in terms of self-efficacy with regard to personal hygiene. It was also associated with female gender, higher age, and lower education. In comparison, planning to frequently wash hands during a severe outbreak was represented by a model including three significant variables and an acceptable discriminative performance (AUC 0.75 (95% CI 0.71;0.80)). Similar to the mild influenza scenario, it was strongly associated with coping appraisal in terms of a high self-efficacy with regard to personal hygiene. The intention was also associated with female gender and being born in the country.

**Table 6:** Simple and multiple logistic regression models of explanatory factors for the intention to wash hands after touching common objects displayed by influenza outbreak scenario.

	Mild influenza scenario		Severe influenza scenario	
	Simple models	Multiple model^a^	Simple models	Multiple model^b^
	OR (95% C.I.)	OR (95% C.I.)	OR (95% C.I.)	OR (95% C.I.)
**Trust in information**				
Trust in official sources	n.s.	n.s.	n.s.	n.s.
**Threat appraisal**				
Perceived personal risk	1.13* (1.01–1.27)	n.s.	1.17* (1.04–1.31)	n.s.
Emotional response to threat	1.15*(1.03–1.29)	n.s.	1.23** (1.10–1.37)	n.s.
Perceived severity of health threat	1.13* (1.02–1.25)	n.s.	1.24** (1.11–1.38)	n.s.
**Coping appraisal**				
General response efficacy†	1.27* (1.09–1.49)	n.s.	1.33** (1.14–1.54)	n.s.
Self-efficacy‡	2.84** (1.89–4.24)	2.54** (1.56–4.13)	2.95** (2.00–4.35)	2.69** (1.65–4.37)
Response costs‡	n.s.	n.s.	n.s.	n.s.
**Sociodemographic characteristics**				
Age	1.02** (1.01–1.03)	1.02* (1.00–1.03)	1.02** (1.01–1.03)	n.s.
Gender (Male = 1;Female = 0)	0.50** (0.34–0.75)	0.50* (0.32–0.78)	0.33** (0.22–0.48)	0.33** (0.21–0.51)
Higher education§ (Yes = 1;No = 0)	n.s.	0.63* (0.41–0.98)	n.s.	n.s.
Born in the country (Yes = 1;No = 0)	n.s.	n.s.	n.s.	0.46* (0.24–0.89)
Living with partner (Yes = 1;No = 0)	n.s.	n.s.	n.s.	n.s.
Living with child (Yes = 1;No = 0)	n.s.	n.s.	n.s.	n.s.
Employment (Yes = 1;No = 0)	0.48** (0.32–0.71)	n.s.	0.47** (0.32–0.69)	n.s.
**Self-rated health (SF-8)**				
Physical Component Summary (PCS)	n.s.	n.s.	0.97* (0.95–0.99)	n.s.
Mental Component Summary (MCS)	n.s.	n.s.	n.s.	n.s.

n.s. = not statistically significant.

* = statistically significant on p = 0.05 level, ** = statistically significant on p = 0.001 level,

a Specificity = 87.8%, Sensitivity = 34.8%, Correctly classified = 69.3%, Nagelkerke R^2^ = 0.176,

b Specificity = 75.0%, Sensitivity = 62.6%, Correctly classified = 69.3%, Nagelkerke R^2^ = 0.259.

† Item specific for personal hygiene.

‡ Item specific for intention to wash hands after touching common objects.

§ Formal education past high school/secondary school.

## Discussion

Despite the fact that the safety of the mass vaccination during the A(H1N1)pdm09 outbreak had been questioned by national mass media in a campaign-like manner, two years after the outbreak every second respondent in a representative sample of the Swedish adult population reported high trust in official information about influenza. While the proportion of persons reporting intentions to improve personal hygiene during influenza outbreaks ranged between 45–85%, the proportion reporting intentions to increase social distancing did not exceed 25%. This pattern can generally be explained by the notion that the initial behavioral changes during an influenza outbreak are more likely to resemble familiar reactions and well-known routines [27], such as increasing personal hygiene, rather than changes that require deductive planning, such as increasing social distancing.

The explanatory models developed in this study showed statistical associations ranging from strong (staying home without being ill) to acceptable (avoiding public transportation and increasing personal hygiene). Among the explanatory factors considered, coping appraisal was the factor most frequently showing associations (as displayed by odds ratios) with the reported intentions. In a validation analysis (data not shown), we fitted each model fully (including all terms in the five explanatory factors categories trust of information, threat appraisal, coping appraisal, sociodemographic factors, and self-rated health) and calculated the proportion of correctly classified cases for these full models for all eight scenarios. Then we left the terms from one of the five categories out separately, and calculated the proportion of correctly classified cases for each of these subset models. We found that the proportion of correctly classified cases without coping appraisal was lower than the corresponding proportion for all full models and lower or equal to the corresponding proportion for 28 of the 32 models excluding one of the other four categories. We interpret these observations combined as indicative evidence that of the explanatory factors considered, coping appraisal was the factor strongest associated with the reported intentions. Analogous to our results, a recent British web-based survey of university employees found that coping appraisal was the principal predictor of variability in protective intentions during pandemics [21], and response costs have been reported as the largest predictor for emergency nurses not reporting to work during an influenza pandemic [28]. A contributing influence to the lesser relative importance of threat appraisal suggested by our results may be a Scandinavian tendency to perceive risks lower than in other countries [29]–[31]. One of the explanations for this tendency is that the media in Scandinavia appear to report more about risks abroad with less attention to risk inside the country [29]. In contrast to our results, self-efficacy during the A(H1N1)pdm09 outbreak in Hong Kong was found to be only weakly associated with social distancing [25]. However, Hong Kong residents are limited in their ability to avoid crowds, and the relatively mild impact of the outbreak could have led to the notion that people saw no reason to jeopardize their economic well-being and curtail other social activities. A socio-geographic theory of protective behaviors during infectious disease outbreaks suggested that efficacy beliefs of Chinese living in the UK and the Netherlands were comparable to those of native UK and Dutch residents during the SARS outbreak in 2003 [32], indicating that country of residence is more important than ethnicity or country and culture of origin in determination of protective behaviors. However, with coordinated regional disease control efforts and increasing influence from social media, this may change.

Gender was the sociodemiographic characteristic that showed the strongest association with the observed variation in reported intentions. As also found in a Norwegian study from the same time period [33], the Swedish women in this study were more disposed to enhance their protective behaviors related to personal hygiene than were men. One explanation of this finding could be an interaction with concerns about the consequences of getting infected. A recent study from the U.S. reported that women were more worried than men about getting seriously ill or even dying during a severe influenza outbreak [34]. However, no gender differences with regard to threat appraisal were reported from the Norwegian study [33]. Originally, we did not include interaction terms in our statistical analyses. A secondary analysis (data not shown) did not reveal any statistically significant interaction between gender and any threat or coping appraisal item such that omitting the interaction from the model would disturb the estimation of the main effects. Therefore, an alternative explanation of our findings is that the female respondents were more disposed to enhance their protective behaviors related to personal hygiene than the male respondents because Swedish women purchase and use hygiene products more often than men [35], and, in consequence, were more confident about the practical handling of handwash and liquid soap. Conversely, men were more inclined to stay home without being ill during influenza outbreaks. This could be explained by the fact that fewer of the employed Swedish men (12%) than women (46%) were at the time of the study working in caring or educational occupations that require physical presence at the workplace, such as nursing, child care, and teaching [36]. In other words, a larger proportion of men could consider the possibility of staying home while continuing to work during an ongoing influenza outbreak, which was not an option for many women. These findings indicate that more research is needed to understand gender-related differences in protective behavior during influenza outbreaks.

The main strengths of this study are its foundation on a current theoretical model [14] and a relatively large representative sample of the Swedish population. However, the study has also important limitations that must be taken into consideration when interpreting the results. The demographic characteristics available may not be the most important factors biasing the results. For instance, it is possible that individuals with low trust in official information about influenza were under-represented, and anxious individuals worrying about disease risks were over-represented, among the participants. Moreover, interpreting cross-sectional data on protective behaviors is difficult because they confound the motivation and accuracy-associated aspects regarding the causal-temporal relationship between perception and behavior [37]. The motivational hypothesis assumes that high perceived risk leads people’s intention to adopt protective behaviors, while the accuracy hypothesis suggests that people who act in a more risky way should also feel more at risk. As an example, individuals having physical contact with many people through their occupation may have been aware of that daily routines are associated with a higher risk for getting infected. Accordingly, a negative correlation may indicate accurate relative risk perceptions, i.e. that people are aware of their risk status [20], [37]. Further longitudinal studies of protective behaviors during influenza outbreaks are thereby warranted [38].

Another limitation is that we assessed self-reported intentions rather than objectively measured behavior. Nevertheless, intentions are a well-validated proxy for behavior predicting a moderate amount (30–42%) of the variance in actual behavior across a wide range of contexts [39], [40]. Moreover, proponents of dual-process health behavior models have suggested that analytic central and emotional-heuristic processes work in concert to select decisions [14], and under certain circumstances emotions may even be the dominant force [41]. While the PMT used in this study does include an emotional component, it still represents a cognitive appraisal model in assuming that cognitive risk assessment determines experience of fear. Such a model is naturally applicable for the study of behaviors aimed at fending off long-term disease, where fear is likely to be less imminent and therefore secondary to more rational reflections about gains and losses related to protective behavior. However, in an acute threat situation, like a severe influenza outbreak, emotional aspects might gain more immediate importance. This would even be more likely during periods of scientific uncertainty, when fewer facts are available. It is in this context interesting to note that coping appraisal in this study was found to be the motivation factor that contributed most to the discriminatory performance despite the fact that threat-affect was included in the general model, although indirectly through cognitive assessment. However, what role affect- or emotion-based judgments play in interaction with threat and coping appraisals is still an issue in need of clarification. Finally, it should be noted that there were relatively small differences between the reported intended behaviors associated to the mild and severe scenarios, respectively. One explanation of this observation can be the fact that the A(H1N1)pdm09 outbreak was relatively mild in Sweden, and that the respondents, wrongfully, related the severe scenario to their recent personal experience rather than the scenario description. However, the lack of difference can also be seen as a sign of its own, i.e. that the Swedish population may not be fully aware of the seriousness of a full influenza pandemic.

Failure to monitor the beliefs and attitudes of the public has recently been identified as a weakness in preparedness strategies against infectious disease outbreaks [42]. We examined how items in a general explanatory model of intended health behavior were associated with personal hygiene and social distancing practices following a questioned mass vaccination campaign against influenza in the Swedish population. We observed a relatively high trust in official recommendations and a higher proportion of intentions to improve personal hygiene than those used to increase social distancing. Among the explanatory factors considered, coping appraisal was the factor most frequently included in models explaining self-reported intentions. Variations in threat appraisal played a smaller role in these models despite the uncertainties surrounding the mass vaccination during the A(H1N1)pdm09 outbreak. The results also show that not just from a third world perspective [43] it is necessary to consider that not all population sub groups have the same predispositions to enact specific behaviors to protect their health. For instance, they suggest that further studies are needed of gender differences in protective behaviors during influenza outbreaks. We conclude that developing interventions that support the general population’s efforts to perform self-protective behaviors during influenza outbreaks and longitudinal studies of such interventions across several influenza seasons are warranted also in European countries.

## Supporting Information

Text S1 **Interview guideline for collection of data on perceptions associated with precautionary behaviors.**
(DOCX)
