# Supplementary material for: Intentions to Perform Non-Pharmaceutical Protective Behaviors during Influenza Outbreaks in Sweden: A Cross-Sectional Study following a Mass Vaccination Campaign
Source: PLoS One. 2014 Mar 7;9(3):e91060. doi: 10.1371/journal.pone.0091060 (PMC3946657; doi:10.1371/journal.pone.0091060)
Supplement: Text S1 — Interview guideline for collection of data on perceptions associated with precautionary behaviors. (DOCX) [file pone.0091060.s001.docx]

**Telephone interview guideline**

*Do consider two scenarios for influenza outbreaks, one scenario with a seasonal influenza outbreak and one with a pandemic influenza outbreak. The personal risk of infection is high in both cases (1 in 3 people are infected). The seasonal influenza infection has moderate health consequences (less than 1 in 1000 infected people dying) and a minor lifestyle impact (services are mainly operating normally). The pandemic influenza infection has serious health consequences (1 in 50-100 infected people dying) and services are no longer being able to operate normally.*

*We are now going to read some statements about attitudes about a flu outbreak and what opportunities people have to protect themselves from being infected. We want you to answer on a scale of 1-7 how well these statements apply to you, where 1 means you do not agree at all and 7 means you agree completely.*

I trust the information on the flu outbreak and what you can do to not get infected if the information comes from public authorities

I think the risk that I suffer from influenza during an outbreak is larger in comparison to others in my age group?

I worry about being hit by the flu:

1. During outbreaks of seasonal influenza
2. In the context of a pandemic influenza outbreak with spread globally

It has serious consequences for me if I suffer from:

1. getting ill with seasonal flu
2. getting ill with pandemic influenza with global spread

I think people in general can protect themselves from the flu by :

1. being more careful with personal hygiene
2. avoiding social contacts

I believe that I can protect myself from the flu by:

1. being more careful with personal hygiene
2. avoiding social contacts

During an influenza outbreak, it would be hard for me to:

1. stay home from work or school without actually being sick
2. getting home and properly use soap and hand sanitiser

*We will now ask some questions about what you would do in connection with an outbreak of seasonal influenza and an outbreak of a severe pandemic with global reach. We want you to answer on a scale of 1-7 how well these statements apply to you, where 1 means you do not agree at all and 7 means you agree completely.*

During an outbreak of seasonal influenza, I would :

Use a liquid soap or hand rub when I wash hands

Wash hands after having touched a handle or buttons in public places

Stay home from work or studies

Avoid travel on public transport

During an outbreak of serious influenza pandemic with global spread, I would :

Use a liquid soap or hand rub when I wash hands

Wash hands after having touched a handle or buttons in public places

Stay home from work or studies

Avoid travel on public transport
